# OsbZIP18, a Positive Regulator of Serotonin Biosynthesis, Negatively Controls the UV-B Tolerance in Rice

**DOI:** 10.3390/ijms23063215

**Published:** 2022-03-16

**Authors:** Yangyang Sun, Bi Wang, Junxia Ren, Yutong Zhou, Yu Han, Shuying Niu, Yuanyuan Zhang, Yuheng Shi, Junjie Zhou, Chenkun Yang, Xuemin Ma, Xianqing Liu, Yuehua Luo, Cheng Jin, Jie Luo

**Affiliations:** 1 College of Tropical Crops, Hainan University, Haikou 570228, China; sunyyhn@hainanu.edu.cn (Y.S.); wongbee@hainanu.edu.cn (B.W.); renjunxiaa@163.com (J.R.); zhouyutong@hainanu.edu.cn (Y.Z.); 15234548597@163.com (Y.H.); 19090102210017@hainan.edu.cn (S.N.); yuanyuan.zhang@hainanu.edu.cn (Y.Z.); shiyuheng@hainanu.edu.cn (Y.S.); sand_zhou@hainanu.edu.cn (J.Z.); liuxq@hainanu.edu.cn (X.L.); lyhhk@163.com (Y.L.); 2 Sanya Nanfan Research Institute of Hainan University, Hainan Yazhou Bay Seed Laboratory, Sanya 572025, China; 3 National Key Laboratory of Crop Genetic Improvement and National Center of Plant Gene Research (Wuhan), Huazhong Agricultural University, Wuhan 430070, China; victoryang@webmail.hzau.edu.cn; 4 Umeå Plant Science Centre, Department of Forest Genetics and Plant Physiology, Swedish University of Agricultural Sciences, 90183 Umeå, Sweden; xuemin.ma@slu.se

**Keywords:** serotonin, UV-B tolerance, basic leucine zipper transcription factor, transcriptional regulation

## Abstract

Serotonin (5-hydroxytryptamine) plays an important role in many developmental processes and biotic/abiotic stress responses in plants. Although serotonin biosynthetic pathways in plants have been uncovered, knowledge of the mechanisms of serotonin accumulation is still limited, and no regulators have been identified to date. Here, we identified the basic leucine zipper transcription factor OsbZIP18 as a positive regulator of serotonin biosynthesis in rice. Overexpression of *OsbZIP18* strongly induced the levels of serotonin and its early precursors (tryptophan and tryptamine), resulting in stunted growth and dark-brown phenotypes. A function analysis showed that OsbZIP18 activated serotonin biosynthesis genes (including *tryptophan decarboxylase 1 (OsTDC1)*, *tryptophan decarboxylase 3* (*OsTDC3*), and *tryptamine 5-hydroxylase* (*OsT5H*)) by directly binding to the ACE-containing or G-box *cis*-elements in their promoters. Furthermore, we demonstrated that *OsbZIP18* is induced by UV-B stress, and experiments using UV-B radiation showed that transgenic plants overexpressing *OsbZIP18* exhibited UV-B stress-sensitive phenotypes. Besides, exogenous serotonin significantly exacerbates UV-B stress of *OsbZIP18*_OE plants, suggesting that the excessive accumulation of serotonin may be responsible for the sensitivity of *OsbZIP18*_OE plants to UV-B stress. Overall, we identified a positive regulator of serotonin biosynthesis and demonstrated that UV-B-stress induced serotonin accumulation, partly in an OsbZIP18-dependent manner.

## 1. Introduction

Serotonin (5-hydroxytryptamine) is a well-known neurotransmitter that controls many critical physiological processes, such as anxiety, mood, and sleep, in humans and animals [1]. Since the first identification of serotonin in plants coming in the legume *Mucuna pruriens* [2], an increasing number of reports have evidenced its wide distribution in plants [3,4]. Similar to the multiple roles in humans and animals, serotonin has also been suggested to be involved in various developmental processes and biotic/abiotic stress responses in plants, such as seed germination, flowering, senescence, shoot branching, root architecture, and protection against pathogens [5,6,7,8,9,10,11,12].

Serotonin biosynthesis occurs from *l*-tryptophan through two enzymatic steps in plants. Tryptophan decarboxylase (TDC) catalyzes the decarboxylation of tryptophan to produce tryptamine, and tryptamine-5-hydroxylase (T5H) hydroxylates the C-5 position of tryptamine to form serotonin [13,14,15]. In rice, three *TDC* genes (*OsTDC1*, *OsTDC2*, and *OsTDC3*) and one *T5H* gene have been identified [6,13,14,16]. In vitro enzymatic assays showed that OsTDC1 and OsTDC3 have high substrate specificity to tryptophan [14]. Overexpression of either *OsTDC1* or *OsTDC3* could significantly increase serotonin levels and results in stunted growth, a dark-brown color, and a low fertility in rice, indicating that serotonin over-accumulation may be deleterious to plants [5,6]. OsT5H, encoding a cytochrome P450 monooxygenase, catalyzes the conversion of tryptamine to serotonin [8,13,17]. In *Sekiguchi lesion* (*sl*) mutants, which lack a P450 monooxygenase, leaves inoculated with the rice blast *Magnaporthe grisea* or rice brown spot disease *Bipolaris oryzae* had enhanced susceptibility to the pathogens but lacked the typical brown material of spot lesions [8,13,18,19].

Although serotonin biosynthesis has been identified in plants, little is known about the molecular mechanisms regulating serotonin accumulation. UV-B radiation (280–315 nm) is not only a potential source of oxidative stress threatening plant growth and development but is also a key environmental signal affecting gene expression, physiology and/or morphology, and the accumulation of plant secondary metabolites. High levels of UV-B cause DNA damage and generate oxidative stress by the formation of reactive oxygen species [20]. In order to cope with UV-B stress, plants produce protective compounds that remodel the metabolism pathway to serve the energy demand under stress. These compounds, especially the metabolites of ascorbic acid and the phenylpropanoid biosynthetic pathway (e.g., flavonoids and hydroxycinnamic acids), accumulate in plants in response to UV-B stress [21,22,23,24]. However, it is unknown whether serotonin-related metabolites are involved in the response to UV-B stress.

Transcription factors (TFs) are the key controllers of signaling regulatory networks in response to abiotic stresses by regulating the expression of target genes. The basic leucine zipper (bZIP) family is one of the largest TF families in plants and it regulates target gene expression directly by binding ‘ACE’ motifs (ACGT-containing elements) in promoters of downstream genes [25]. Based on the characteristics of bZIP structural homology, 75 bZIPs in *Arabidopsis* and 89 bZIPs in rice were divided into 13 groups (A–L and S) and 11 groups (I–XI), respectively [26,27]. Some bZIP members have been shown to be involved in regulating plant growth and development and playing important roles in the responses to various abiotic stresses [28,29,30,31,32,33,34,35,36,37,38,39,40,41,42]. AtHY5, a member of the *Arabidopsis* S1-bZIP group, is a central regulator of UV-B protection that promotes photomorphogenesis downstream to multiple photoreceptors and the expression of light-induced genes [43]. In rice, three homologs of AtHY5 (*OsbZIP01*, *OsbZIP18*, and *OsbZIP48*) have been identified [44]. OsbZIP48 could complement the *athy5* mutant with respect to hypocotyl elongation growth in the light and negatively regulated plant height by binding to G-box elements in *OsKO2* (*ent-kaurene oxidase 2*), the promoter of the gibberellin genes [44]. Further study showed that OsbZIP48 positively regulated flavonoid accumulation and UV-B resistance in rice [45]. Recently, Sun et al. [46,47] revealed that OsbZIP18 positively regulates branched-chain amino acid accumulation in rice leaves by binding directly to the ACE and C-box *cis*-elements in the promoters of the biosynthetic genes *branched-chain aminotransferase1* (*OsBCAT1*) and *OsBCAT2*. However, the physiological function of *OsbZIP18* is unclear, and its role in the response to abiotic stresses has not been explored in rice.

The aim of this study was to investigate the molecular mechanism of the leucine zipper (bZIP) transcription factor *OsbZIP18* in regulating serotonin biosynthesis in rice. We demonstrated that OsbZIP18 is a positive regulator of serotonin that negatively controls UV-B tolerance in rice. Overexpression of *OsbZIP18* significantly increased serotonin levels, resulting in stunted growth and dark-brown phenotypes. A function analysis showed that OsbZIP18 activated serotonin biosynthesis genes (*OsTDC1*, *OsTDC3*, and *OsT5H*) by directly binding to their promoters. Furthermore, we demonstrated that UV-Binduced serotonin-related metabolite accumulation in an OsbZIP18-dependent manner.

## 2. Results

### 2.1. UV-B Radiation Stimulates Endogenous Serotonin Levels in Rice

**Figure 1:**
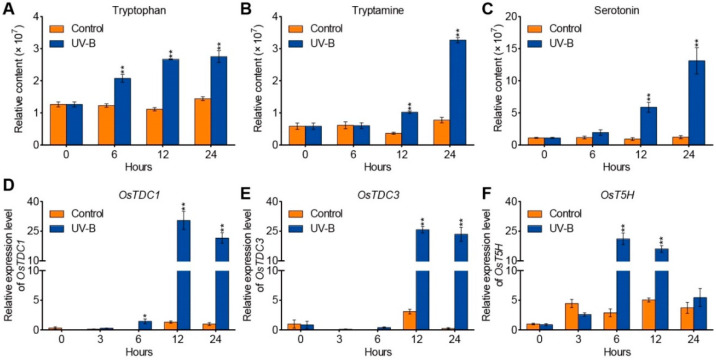
Serotonin biosynthesis in Zhonghua11 (ZH11) increased under UV-B radiation. (**A**–**C**) Accumulation of tryptophan (**A**), tryptamine (**B**), and serotonin (**C**) in the leaves of ZH11 under UV-B radiation. (**D**–**F**) Expression analysis of serotonin biosynthesis genes *OsTDC1* (**D**), *OsTDC3* (**E**), and *OsT5H* (**F**) under control and UV-B radiation conditions. The relative expression levels were normalized to those of *ubiquitin*. Two-week-old seedlings were subjected to UV-B for 0, 3, 6, 12, and 24 h. The data are means ± SD of three biological replicates. Asterisks indicate a significant difference between the control and UV-B treatments at individual time points (* *p* < 0.05 and ** *p* < 0.01, Student’s *t*-test).

To examine whether UV-B radiation would affect the primary and secondary metabolites in rice, we assayed a metabolic profiling analysis in seedlings (ZH11) under UV-B stress using a widely targeted liquid chromatography-mass spectrometry (LC-MS) method [48]. Under the UV-B treatment, large amounts of serotonin and its early precursors (tryptophan and tryptamine) were rapidly produced, suggesting that the biosynthesis of serotonin from tryptophan was induced by UV-B radiation (Figure 1A–C). Furthermore, the transcription levels of three serotonin biosynthesis genes (*OsTDC1*, *OsTDC3*, and *OsT5H*) were upregulated in response to UV-B treatment in rice (Figure 1D–F). These findings indicate that UV-B radiation enhanced the endogenous serotonin contents in rice.

### 2.2. UV-B Radiation Alters the Expression Levels of OsbZIP01, OsbZIP18, and OsbZIP48 in Rice

**Figure 2:**
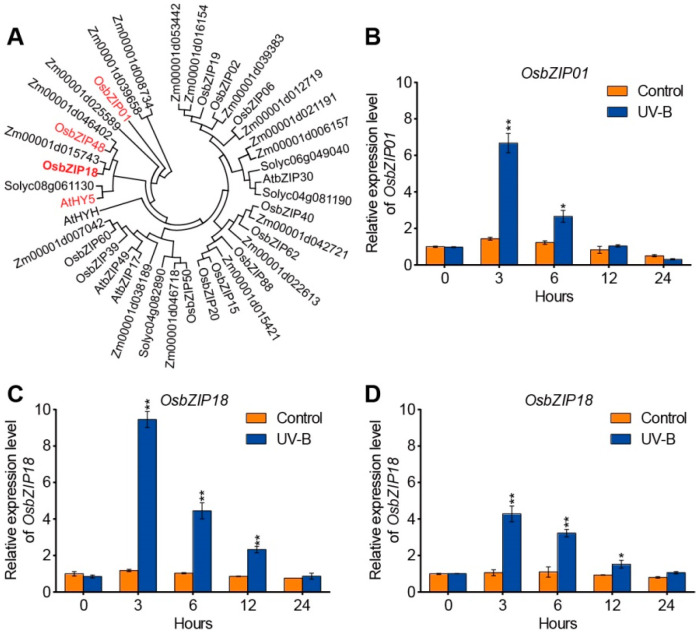
Effect of UV-B radiation on the transcript levels of rice *HY5* genes. (**A**) The phylogenetic tree of HY5 protein in *Arabidopsis*, rice, maize, and tomato. The values of bootstraps from 1000 replicates are indicated. (**B**–**D**) Expression analysis of *OsbZIP01* (**B**), *OsbZIP18* (**C**), and *OsbZIP48* (**D**) under control and UV-B radiation. The relative expression levels were normalized to those of *ubiquitin* and were quantified by RT-qPCR. Two-week-old seedlings were subjected to UV-B for 0, 3, 6, 12, and 24 h. Os, *Oryza sativa*; At, *Arabidopsis thaliana*; Zm, *Zea mays*; Solyc, *Solanum lycopersicum*. The data are means ± SD of three biological replicates. Asterisks indicate a significant difference between the control and UV-B treatments at individual time points (* *p* < 0.05 and ** *p* < 0.01, Student’s *t*-test).

The UV-B radiation experiments suggested a positive regulatory role of UV-B in serotonin biosynthesis gene regulation (Figure 1D–F). Since *Arabidopsis* bZIP transcription factor *AtHY5* is a crucial gene that positively regulates a subset of gene transcription in response to UV-B radiation [43,49,50,51,52], we determined whether its orthologs in rice were functionally similar to *AtHY5*. The phylogenetic analysis showed that there were three homologs of AtHY5 (*OsbZIP01*, *OsbZIP18*, and *OsbZIP48*) in rice, exhibiting 55%, 66%, and 57% amino acid sequence identity to AtHY5, respectively (Figure 2A and Figure S1).

Reverse transcription-quantitative PCR (RT-qPCR) showed that *OsbZIP01*, *OsbZIP18*, and *OsbZIP48* were all induced by UV-B radiation, with ~6-, 10-, and 4-fold increases in leaves at 3 h following the UV-B radiation treatment, respectively (Figure 2B–D). Among those three, the *OsbZIP18* expression level showed the greatest (~10-fold) increase at 3 h after UV-B treatment (Figure 2C). Therefore, we selected *OsbZIP18* for further analysis.

### 2.3. OsbZIP18 Positively Regulates Serotonin Biosynthesis in Rice

**Figure 3:**
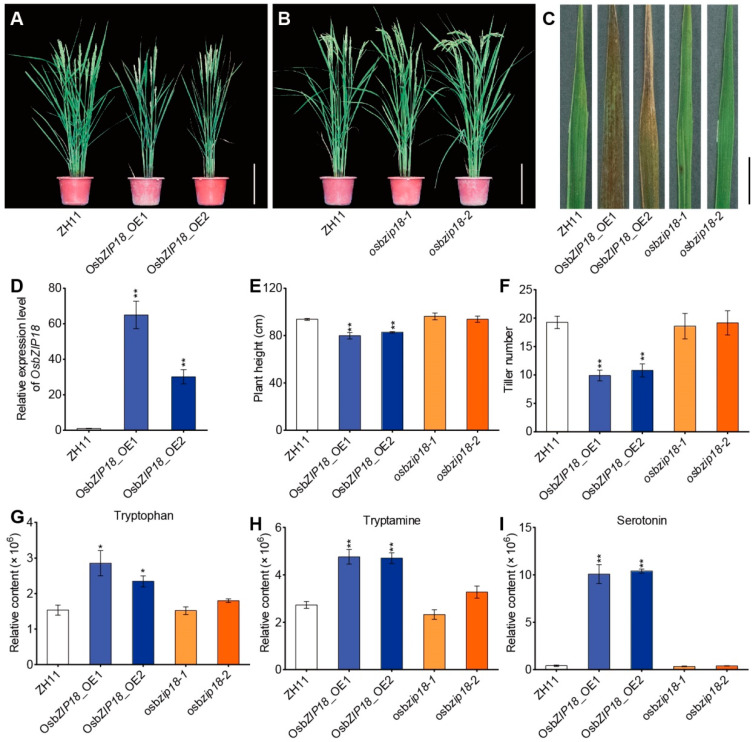
Appearance and serotonin biosynthesis of *OsbZIP18* transgenic lines and their wild type (ZH11). (**A**,**B**) Phenotype of two *OsbZIP18*-overexpression lines (*OsbZIP18*_OE1 and *OsbZIP18*_OE2), two *osbzip18* knockout mutants (*osbzip18-1* and *osbzip18-2*), and the wild-type rice (ZH11). The plants were cultivated in a paddy field in a tropical area (Lingshui, Hainan). (**C**) Appearance of the dark-brown pigmentation in the mature leaves. (**D**) Relative expression levels of *OsbZIP18* in the leaves of the wild type (ZH11) and two independent overexpression lines. The *ubiquitin* gene was used as the endogenous control. (**E**,**F**) The plant height (**E**) and tiller number (**F**) in (**A**,**B**). (**G**–**I**) The accumulation of tryptophan (**G**), tryptamine (**H**), and serotonin (**I**) in the leaves. All data were compared with ZH11. Scale bars = 20 cm for (**A**,**B**) and 1 cm for (**C**). The *p*-value was calculated using Tukey’s test; *n* = 6 (**E**,**F**), *n* = 3 (**D**,**G**–**I**). * *p* < 0.05 and ** *p* < 0.01.

To evaluate the relationships between *OsbZIP18* and serotonin, we generated *OsbZIP18* overexpression (*OsbZIP18*_OE) plants in the Zhonghua11 background. The expression level of *OsbZIP18* in the leaves was checked and was indeed significantly higher than in the wild type (Figure 3D). Two *osbzip18* mutants were obtained using the CRISPR-*Cas9* method as previously described [46]. When grown in a paddy field at Lingshui (Hainan), *OsbZIP18*_OE plants showed pleiotropic growth disorders, including a significant decrease in the plant height and tiller number and severe dark-brown pigmentation in the mature leaves (Figure 3A,C,E,F). No obvious effects on plant height, tiller number, or pigmentation of the *osbzip18* mutants compared with ZH11 were found at the reproductive stage (Figure 3B,C,E,F).

Our metabolic profiling analysis using LC-MS showed that all *OsbZIP18*_OE lines grown in the field accumulated significantly elevated levels of serotonin and its precursors (tryptophan and tryptamine) in the leaves compared with wild-type plants (Figure 3G–I). It is worth noting that there was a massive 26-fold difference in the leaf serotonin contents between the *OsbZIP18*_OE plants and the wild type (Figure 3G–I), which suggests that OsbZIP18 could promote serotonin biosynthesis in rice. However, no significant differences in the levels of serotonin and its precursors (tryptophan and tryptamine) were found between the *osbzip18* mutants and the wild-type plants (Figure 3G–I). Taken together, these results suggest that the serotonin accumulation in transgenic rice by overexpressing *OsbZIP18* could lead to a dark-brown pigmentation phenotype and stunted growth.

### 2.4. OsbZIP18 Directly Binds to the Promoter of Serotonin Biosynthesis Genes OsTDC1, OsTDC3, and OsT5H

**Figure 4:**
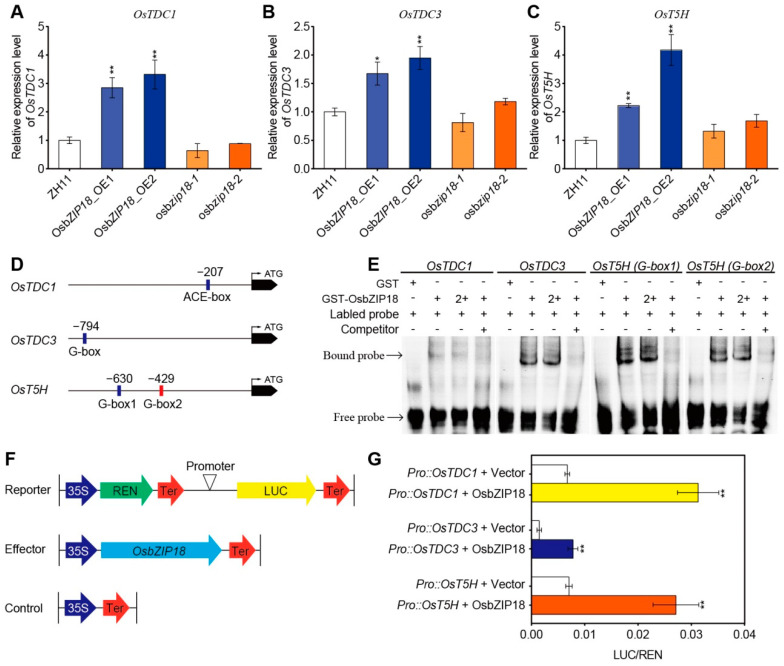
OsbZIP18 directly binds to the ACE/G-box *cis*-elements of serotonin biosynthesis genes and positively regulates their expression. (**A**–**C**) The expression level of serotonin biosynthesis genes *OsTDC1* (**A**), *OsTDC3* (**B**), and *OsT5H* (**C**) in the leaves of the indicated plants. The relative expression levels were normalized to those of *ubiquitin*. All plants were grown in a paddy field. (**D**) Diagram of the *OsTDC1*, *OsTDC3*, and *OsT5H* promoter regions, showing the relative positions of the ACE/G-box *cis*-elements (blue and green rectangles). (**E**) Electrophoretic mobility shift assay (EMSA) assay, showing the OsbZIP18 recombinant proteins bound to the ACE/G-box *cis*-elements in the *OsTDC1*, *OsTDC3*, and *OsT5H* promoters. (**F**) Schematic diagram of the effector and reporter plasmids used in the transient assay in leaf epidermal cells of *N. benthamiana*. Ren, Renilla luciferase; LUC, firefly luciferase. (**G**) OsbZIP18 activates the transcription of *OsTDC1*, *OsTDC3*, and *OsT5H*. *N. benthamiana* leaves were infiltrated with different combinations of effectors and reporters. The LUC activity was normalized to the REN activity as an internal control. The *p*-value was calculated using Tukey’s test, *n* = 3. * *p* < 0.05 and ** *p* < 0.01.

Next, we investigated how OsbZIP18 promotes serotonin biosynthesis at the molecular level. As serotonin biosynthesis begins with tryptophan and consists of two enzymatic steps, we hypothesized that OsbZIP18 may regulate serotonin biosynthesis by directly or indirectly modulating the expression of serotonin biosynthesis genes (*OsTDC* and *OsT5H*) involved in these two enzymatic steps. To verify this hypothesis, we first examined the transcription levels of serotonin biosynthesis genes in the *OsbZIP18* transgenic plants. RT-qPCR experiments revealed that the expression of *OsTDC1*, *OsTDC3*, and *OsT5H* were upregulated in the leaves of the *OsbZIP18*_OE lines compared with the wild-type (Figure 4A–C). However, the expressions of these three serotonin biosynthesis genes were not altered significantly in the *osbzip18* mutants (Figure 4A–C), which is consistent with the levels of serotonin and its precursors in the *osbzip18* mutants as compared with the wild-type plants (Figure 3G–I).

Since *OsTDC1*, *OsTDC3*, and *OsT5H* were shown to be upregulated in the *OsbZIP18*_OE plants (Figure 4A–C), promoter analyses of *OsTDC1*, *OsTDC3*, and *OsT5H* were performed manually, as they could be direct targets of OsbZIP18. The analyses revealed that there was one or two ACE-containing or G-box (CACGTG) elements within the 1-kb region upstream of the transcription start site of the *OsTDC1*, *OsTDC3*, and *OsT5H* genes (Figure 4D), which have been identified as the essential binding motif of HY5 in *Arabidopsis* [43,46,53,54]. As such, we then investigated whether OsbZIP18 could bind to the promoters of *OsTDC1*, *OsTDC3*, and *OsT5H* in vitro. An EMSA showed that OsbZIP18 bound to the ACE element 207 bp upstream from the transcription start site of *OsTDC1* to the G-boxes 794 bp upstream from the transcription start site of *OsTDC3* and 630 bp/429 bp upstream from the transcription start site of *OsT5H* (Figure 4E). The binding of OsbZIP18 to the G-boxes in the promoters of *OsTDC3* and *OsT5H* was stronger than to the ACE element in *OsTDC1* (Figure 4E). These results suggest that OsbZIP18 regulates the expression of serotonin biosynthesis genes (including *OsTDC1*, *OsTDC3*, and *OsT5H*) by directly binding to their promoters.

To further confirm the results, we cotransfected the effector OsbZIP18 (35S::OsbZIP18) with the reporter containing the *OsTDC1*, *OsTDC3*, or *OsT5H* promoter driving luciferase (Pro *OsTDC1*: LUC, Pro *OsTDC3*: LUC, or Pro *OsT5H*: LUC) in leaf epidermal cells of *N. benthamiana*, and the relative luciferase (LUC) activity was measured (Figure 4F). The results showed that co-expression with OsbZIP18 (35S::OsbZIP18) significantly increased the luciferase activity of Pro *OsTDC1*: LUC, Pro *OsTDC3*: LUC, and Pro *OsT5H*: LUC (Figure 4G), indicating that OsbZIP18 functions as a transcriptional activator of *OsTDC1*, *OsTDC3*, and *OsT5H* expression.

### 2.5. OsbZIP18_OE Plants Exhibit UV-B Stress-Sensitive Phenotypes

**Figure 5:**
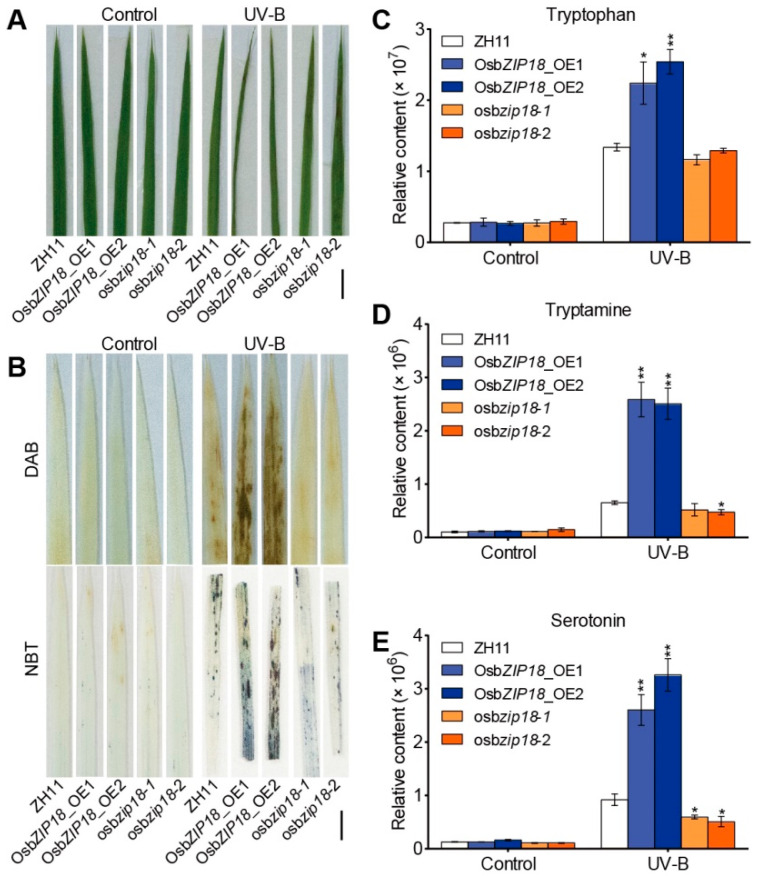
The phenotype and serotonin biosynthesis of the *OsbZIP18* transgenic lines and their wild type (ZH11) under control and UV-B radiation. (**A**) Phenotype comparison of the leaf curl under the control or UV-B stress conditions. (**B**) DAB and NBT staining were used to assess the accumulation of H_2_O_2_ and O_2_^−^, respectively. Fourteen-day-old seedlings grown in a light incubator were treated with or without UV-B for 36 h before staining. (**C**–**E**) The accumulation of tryptophan (**C**), tryptamine (**D**), and serotonin (**E**) in the leaves under UV-B radiation. All data were compared with ZH11. Scale bars = 5 mm for (**A**,**B**). The *p*-value was calculated using Tukey’s test, *n* = 3. * *p* < 0.05 and ** *p* < 0.01.

To further investigate the role of OsbZIP18 in UV-B stress responses, the leaf phenotypes and ROS accumulation in leaves were examined. Fourteen-day-old seedlings of the *OsbZIP18* transgenic lines and the wild type plants grown in a climate chamber with white light were transferred to a UV-B-radiating (11.06 KJ m^−2^ d^−1^) chamber without white light. Under white light conditions, we observed no significant difference in the leaf phenotypes among the *OsbZIP18*_OE lines, *osbzip18* mutants, and wild type plants (Figure 5A). However, under UV-B stress, the leaf growth of the *OsbZIP18*_OE plants was more withered than that of the wild type (Figure 5A). The staining of leaves with 3,3′-diaminobenzidine (DAB) and nitrotetrazolium blue chloride (NBT) revealed that the accumulation of H_2_O_2_ and O_2_^−^ was higher in the *OsbZIP18*_OE plants but lower in the *osbzip18* mutants under UV-B treatment compared to the wild type (Figure 5B). These results indicate that OsbZIP18 negatively regulates the UV-B tolerance in rice.

Subsequently, we compared the metabolic profiles of the *OsbZIP18*_OE lines, *osbzip18* mutants, and wild-type plants using LC-MS. Under white light conditions, there was no significant difference in the levels of serotonin and its precursors (tryptophan and tryptamine) between the *OsbZIP18* transgenic lines and the wild type (Figure 5C–E). However, in the presence of UV-B radiation, the accumulation of tryptophan, tryptamine, and serotonin were significantly induced in all plants (Figure 5C–E). Compared to the wild type, the *OsbZIP18*_OE plants accumulated significantly higher contents of tryptophan, tryptamine, and serotonin, whereas the *osbzip18* mutants reduced the contents of serotonin compared with the wild-type plants (Figure 5C–E). Together, these results suggest that UV-B stress induced serotonin accumulation, partly in an OsbZIP18-dependent manner.

### 2.6. Serotonin Increases the UV-B Stress Sensitivity of OsbZIP18_OE Plants

**Figure 6:**
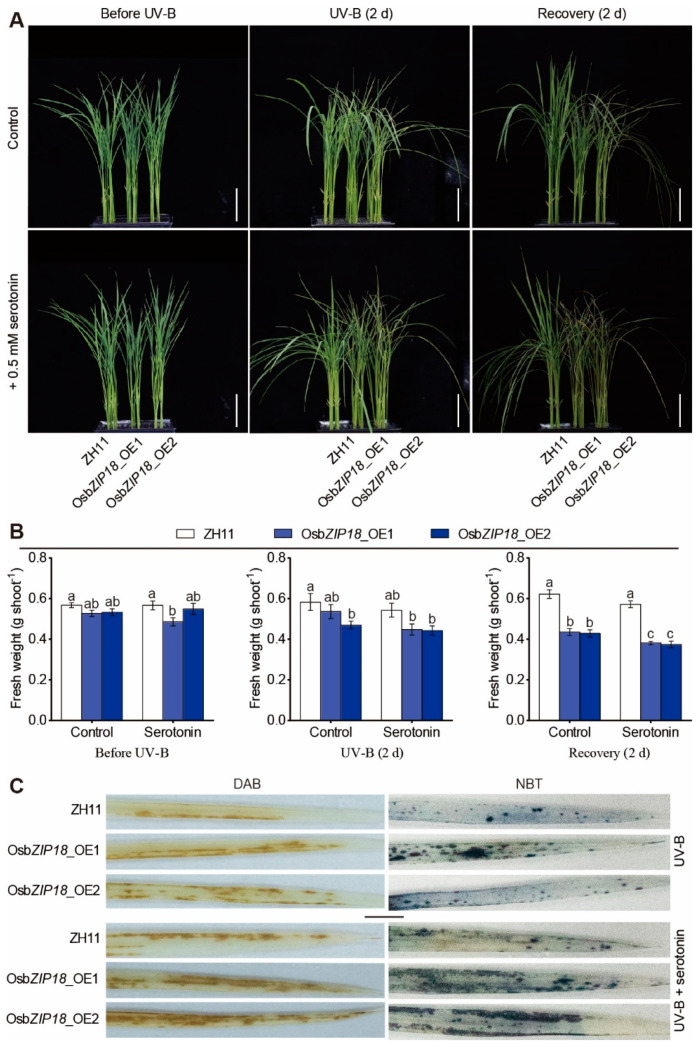
Serotonin increases the UV-B stress-sensitive phenotypes of the *OsbZIP18*-overexpression plants. (**A**) Phenotype comparison of the *OsbZIP18*-overexpression plants (*OsbZIP18*_OE1 and *OsbZIP18*_OE2) before UV-B treatment, after UV-B treatment for 2 d, and after recovery from the UV-B treatment. The plants were grown in a nutrient solution with or without 0.5 mM serotonin. The plants were pretreated with 0.5 mM serotonin for 24 h before UV-B treatment. Scale bars = 5 cm. (**B**) The fresh weight of shoots in (**A**). The data are means ± SD of nine biological replicates. Different letters indicate a significant difference at *p* < 0.05. (**C**) Accumulation of ROS in the leaves treated with UV-B or UV-B + serotonin for 36 h. Scale bars = 5 mm.

To test whether the UV-B stress-sensitive phenotype of *OsbZIP18*_OE plants was caused by an increase in the amount of serotonin, we examined whether the addition of serotonin to the culture solution could exacerbate the UV-B stress-sensitive phenotype of *OsbZIP18*_OE plants. Under white light conditions, the addition of serotonin to the culture solution caused no phenotypic alterations in the *OsbZIP18*_OE lines compared with the wild-type plants (Figure 6A,B). However, in the presence of UV-B radiation, the addition of 0.5 mM serotonin to the culture solution exacerbated the UV-B stress-sensitive phenotype of the *OsbZIP18*_OE plants (Figure 6A,B). Under UV-B stress, the fresh weight of shoots in the *OsbZIP18*_OE plants was lower than that in the wild type plants, which was further aggravated by adding exogenous 0.5 mM serotonin (Figure 6B). Furthermore, the staining of leaves with DAB and NBT revealed that the ROS accumulation in the *OsbZIP18*_OE plants was further aggravated by the exogenous serotonin treatment compared to the wild type (Figure 6C).

**Figure 7:**
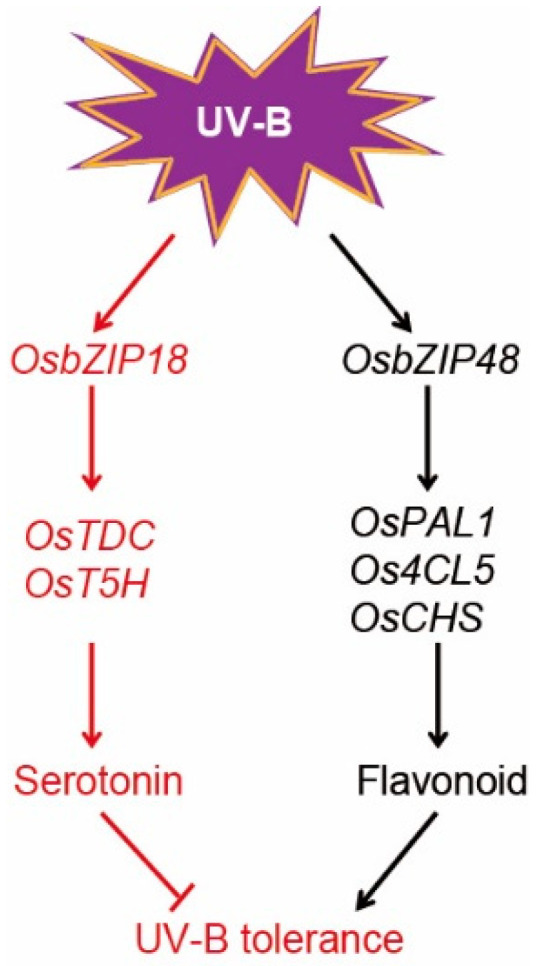
A proposed working model of the role of OsbZIP18 in the regulation of serotonin biosynthesis and UV-B tolerance.

We also examined the effect of 0.5 mM serotonin on the *osbzip18* mutants in the presence or absence of UV-B radiation. Under white light conditions, we observed no differences between the *osbzip18* mutants and wild type plants upon the addition of serotonin (Supplementary Figure S2A,B). In contrast, in the presence of UV-B radiation and no serotonin, *osbzip18* mutants showed more UV-B-tolerant phenotypes than the wild-type plants, including increased fresh weight and decreased ROS accumulation (Supplementary Figure S2A,B). However, after supplying the plants with exogenous serotonin, the *osbzip18* mutants and the wild type showed similar phenotypes (Supplementary Figure S2). The fresh weight and ROS accumulation of the *osbzip18* mutants were almost rescued to the level of the wild-type plants (Supplementary Figure S2B,C). Taken together, these results indicate that the plant serotonin levels determined by OsbZIP18 play an important role in the UV-B stress response (Figure 7).

## 3. Discussion

Serotonin, a natural bioactive compound, which is widespread in plants, is accumulated during drought, salinity, chilling, and pathogen attack conditions [4,7,55,56], suggesting that it plays an important role in the responses to biotic/abiotic stresses. Despite its widespread distribution and activity, there are many questions that remain unanswered about the role of serotonin in plants, including the molecular mechanism of serotonin in response to biotic/abiotic stress.

In this study, we demonstrated that serotonin and its precursors (tryptophan and tryptamine) are responsive to UV-B stress (Figure 1A–C). In the different species studied so far, many structural genes responsible for serotonin levels have been identified. For example, the expression levels of *SlTDC1* and *SlT5H* are related with the serotonin contents in tomato [57], and the overexpression of *SlTDC1* in *Arabidopsis* significantly increased serotonin levels [58]. In contrast with the previously published observations in tomato, we identified a transcriptional regulator, OsbZIP18, which is responsible for the serotonin levels in rice. 

A recent study demonstrated that OsbZIP48 positively regulated flavonoid accumulation and UV-B resistance in rice [45]. In contrast, our results showed that OsbZIP18 positively regulated serotonin biosynthesis and negatively controlled UV-B tolerance (Figure 7). OsbZIP01, OsbZIP18, and OsbZIP48 exhibit high amino acid sequence similarity to *Arabidopsis* HY5, which is the central transcription factor that acts downstream of the UV-B photoreceptor UV RESISTANCE LOCUS8 (UVR8) and promotes UV-B-induced photomorphogenesis [50]. These results indicated that the three *OsbZIP* genes of rice may be functional orthologues of AtHY5. As predicted, OsbZIP48 was shown to be able to complement the *athy5* mutant with respect to hypocotyl elongation growth in the light [44], further demonstrating that OsbZIP48 is a functional orthologue of AtHY5. Transgenic plants overexpressing either *OsbZIP48* or *OsbZIP18* had significantly reduced heights. However, the overexpression of *OsbZIP18* also exhibited a decreased tiller number and severe dark-brown pigmentation in the mature leaves, which were not present in the *OsbZIP48*_OE plants. These results suggest that OsbZIP18 and OsbZIP48 have distinct functions. 

Previous studies showed that serotonin over-accumulation in *OsTDC1* and *OsTDC3* overexpression plants resulted in a dark-brown phenotype and stunted growth [6,59]. Similarly, overexpression of *OsbZIP18* greatly induced serotonin levels and resulted in stunted growth and a dark-brown phenotype in the mature leaves (Figure 3A,C,E–I). The dark-brown pigmentation and higher serotonin levels were observed in the mature leaves of the *OsbZIP18*_OE plants grown in high irradiation areas (Lingshui, Hainan) but not in the leaves of seedlings grown in a climate chamber (120 µmol m^−2^ s^−1^ white light, 16 h day and 8 h night), suggesting that OsbZIP18-mediated regulation of serotonin levels might depend on environment conditions. Furthermore, we showed that OsbZIP18 positively regulated serotonin accumulation by directly binding and activating the serotonin biosynthesis genes *OsTDC1*, *OsTDC3*, and *OsT5H* (Figure 4), suggesting that the dark-brown and stunted growth phenotype of the *OsbZIP18*_OE plants may be caused by excessive serotonin accumulation. To the best of our knowledge, OsbZIP18 is the first regulator of serotonin accumulation identified in plants.

Exogenously added serotonin induces cell death in rice suspension cell cultures and induces brown pigmentation when exposed to UV light [13]. Our results showed that transgenic plants overexpressing *OsbZIP18* greatly induced serotonin levels and exhibited UV-B stress-sensitive phenotypes (Figure 5), suggesting that excessive serotonin accumulation may be responsible for the sensitivity of *OsbZIP18*_OE plants to UV-B stress. In the presence of UV-B radiation and no serotonin, overexpression of *OsbZIP18* significantly reduced UV-B tolerance, causing growth inhibition and a severe dark-brown pigmentation (Figure 6), whereas knocking out *OsbZIP18* enhanced UV-B tolerance compared to the wild-type rice (Supplementary Figure S2). However, after exogenously supplying serotonin, the *osbzip18* mutants showed similar phenotypes to the wild-type rice (Figure 6), and the UV-B stress-sensitive phenotype of the *OsbZIP18*_OE lines was significantly aggravated (Supplementary Figure S2). Therefore, we speculate that an excessive accumulation of serotonin is detrimental to plant resistance to UV-B stress. However, the mechanism by which serotonin regulates UV-B tolerance in rice remains to be further explored.

Taken together, we demonstrated that UV-B is a key environmental factor in the regulation of serotonin biosynthesis, and UV-B stress induced serotonin accumulation, partly in an OsbZIP18-dependent manner (Figure 7). Thus, we provide useful information to uncover the regulatory mechanisms between the transcription factor and serotonin biosynthesize genes to reveal how plants respond and adapt to UV-B stress.

## 4. Materials and Methods

### 4.1. Plant Materials and Growth Conditions

To generate the overexpression construct, the full-length coding sequence of *OsbZIP18* was amplified using rice (*O. Sativa* L. ssp. *japonica* cv. Nipponbare) total cDNA as a template, and its primers were designed based on the sequence of *Os02g0203000* from the NCBI (Available online: https://www.ncbi.nlm.nih.gov/ 25 September 2020). The entry clone was obtained through recombination of the PCR product with pDONR207 (Invitrogen, Carlsbad, CA, USA). Error-free clones were then introduced into the destination vector pJC034 to produce expression vectors by LR recombination [60], which were introduced into *Agrobacterium tumefaciens* EHA105 and were transformed into calli derived from ZH11 (*O. sativa* L. ssp. *japonica* cv. Zhonghua 11) by an *Agrobacterium*-mediated transformation [61]. The *osbzip18* mutants were obtained using the CRISPR-*Cas9* method as previously described [46]. The primer sequences used to generate the construct are listed in Table S1.

For field trials, plants were grown in high-irradiation areas (Lingshui, Hainan Province, China; longitude 110°11′ E, latitude 18°30′ N) with routine management. Hydroponic experiments were performed in a climate chamber (120 µmol m^−2^ s^−1^ white light, 16 h day and 8 h night) using a standard rice culture solution as described previously [60], and the nutrient solution was renewed every 3 d.

### 4.2. UV-B and Exogenous Serotonin Treatment

Seeds of ZH11, *OsbZIP18*_OE1, *OsbZIP18*_OE2, *osbzip18-1*, and *osbzip18-2* were germinated for 4 d at 37 °C on filter paper soaked with distilled water. After germination, the seeds were respectively transferred to a plastic container for hydroponic culture in a climate chamber with a white light intensity of 120 µmol m^−2^ s^−1^ under a 16 h day and 8 h night regime (28 °C). UV-B treatments were performed using UV fluorescent lamps as described previously [45]. Briefly, seedlings were placed in a UV-B-radiating chamber (Philips, Netherlands, TL8W/302 nm narrowband UV-B tube, fluence 11.06 KJ m^−2^ d^−1^) without white light for the indicated times. The control group (−UV-B) was exposed to 120 μmol m^−2^ s^−1^ white light. The UV-B intensity was measured using a UV radiometer with the UV-295 detector from the photoelectric instrument factory of Beijing Normal University, China. After the treatment, the seedlings were returned to the climate chamber for recovery in long-day conditions (120 µmol m^−2^ s^−1^ white light, 16 h day and 8 h night).

For serotonin treatments, ten-day-old seedlings of ZH11, OsbZIP18_OE1, and OsbZIP18_OE2 (or ZH11, *osbzip18-1*, and *osbzip18-2*, 16 plants each) were pretreated with 0.5 mM serotonin for 24 h in a climate chamber (120 µmol m^−^^2^ s^−^^1^ white light) and then treated with UV-B radiation (11.06 KJ m^−2^ d^−1^) for 2 d as mentioned above. The control group was grown in normal nutrient solution (without exogenous serotonin).

### 4.3. Measurement of Tryptophan, Tryptamine, and Serotonin

The content of tryptophan, tryptamine, and serotonin was determined using a liquid chromatography-tandem mass spectrometry (LC-MS/MS) system as previously described [48]. The freeze-dried samples were crushed using a mixer mill (MM 400; Retsch, Haan, Germany) with zirconia beads for 1 min at 30 Hz, and 0.1 g of the dry powder was extracted overnight at 4 °C with 1 mL of 70% aqueous methanol containing 0.1 mg/L lidocaine (internal standard). Following centrifugation at 10,000× *g* for 10 min, the lipid-soluble extracts were absorbed, and 0.4 mL of each extract was mixed and filtered (SCAA-104, 0.22 μm pore size; Angel, Shanghai, China) before the LC-MS analysis.

The targeted metabolic profiling analysis was conducted using scheduled multiple reaction monitoring (MRM) via an LC-ESI-QQQ-MS/MS system (LCMS-8060, SHIMADZU, Kyoto, Japan). The UPLC (Shim-pack UFLC SHIMADZU CBM30A system) conditions were as follows: column, shim-pack GISS C18 (pore size 1.9 μm, dimensions 2.1 × 100 mm); solvent system, water (0.04% acetic acid), acetonitrile (0.04% acetic acid); gradient program, 95:5 *v*/*v* at 0 min, 5:95 *v*/*v* at 12.0 min, 5:95 *v*/*v* at 13.2 min, 95:5 *v*/*v* at 13.3 min, 95:5 *v*/*v* at 15.0 min; flow rate, 0.40 mL min^−1^; temperature, 40 °C; injection volume: 2 μL. The ESI source operation parameters were as follows: nebulizing gas flow, 3 L min^−1^; heating gas flow, 10 L min^−1^; interface temperature, 500 °C; DL temperature, 250 °C; heat block temperature, 400 °C; drying gas flow, 10 L min^−1^. The recorded data were processed with LabSolutions 5.91 software.

### 4.4. RNA Extraction and Expression Analyses

Reverse transcription-quantitative PCR (RT-qPCR) was performed using total RNA extracted with an RNA extraction kit (TRIzol reagent; Invitrogen, Carlsbad, CA, USA) according to the manufacturer’s instructions. Here, 3 μg of RNA was used to synthesize the first-strand cDNAs in 20 μL of the reaction mixture using the EasyScript One-Step gDNA Removal and cDNA Synthesis SuperMix (TransGen, Beijing, China). Quantification of transcript abundance was performed using the SYBR Premix Ex Taq kit (TaKaRa, Tokyo, Japan) on the ABI 7500 Real-Time PCR system (Applied Biosystems, Foster City, CA, USA). The expression levels were normalized to the expression of the rice *UBIQUITIN* gene. All of the RT-qPCR analyses were performed for three biological replicates. The primers for RT-qPCR are listed in Table S1.

### 4.5. Phylogenetic Analysis

The protein sequences of HY5 in *Arabidopsis*, rice, maize, and tomato were obtained from the NCBI (Available online: https://www.ncbi.nlm.nih.gov/ 25 September 2020) and aligned using the CLUSTALW (v.1.83) program. A neighbor-joining tree was constructed using the aligned full-length amino acid sequences (MEGA7, Available online: http://megasoftware.net/ 25 September 2020). Bootstrap values were estimated (with 1000 replicates) to assess the relative support for each branch.

### 4.6. DAB and NBT Staining

The nitrotetrazolium blue chloride (NBT) (Sangon Biotech, Shanghai, China) and diaminobenzidine (DAB) (Sangon Biotech, Shanghai, China) staining assays were performed according to the method as previously described with a slight modification [45]. Two-week-old seedlings were treated with or without UV-B radiation for 36 h. For the DAB staining assays, plant leaves were vacuum infiltrated for 30 min and then stained with 1% DAB (*w*/*v*) for 24 h (pH = 3.8). For the NBT staining assays, the leaves were vacuum infiltrated for 30 min and then stained for 24 h with 0.1% NBT (*w*/*v*) and 10 mM sodium azide in 10 mM potassium phosphate buffer (pH = 7.6). To remove chlorophylls, all stained leaves were transferred to 95% ethanol and incubated at 80 °C for 15 min.

### 4.7. Electrophoretic Mobility Shift Assay

The full cDNA of *OsbZIP18* from Nipponbare were cloned into the pGEX-6p-1 expression vector (Novagen, San Diego, CA, USA) to produce the GST-OsbZIP18 vector and were then introduced into and expressed in *E. coli* BL21 (DE3) cells (Novagen, San Diego, CA, USA). The *E. coli* cells were induced with 0.1 mM isopropyl-*β*-D-thiogalactoside (IPTG) (Sangon Biotech, Shanghai, China) and were grown continually for 16 h at 20 °C. The GST-OsbZIP18 recombinant proteins were purified using Glutathione Sepharose 4B beads (GE Healthcare Biosciences, Uppsala, Sweden). The 50 bp probes containing the G-box motif were synthesized as forward and reverse strands and labeled with FAM. An electrophoretic mobility shift assay was performed using a LightShift chemiluminescent EMSA kit (Thermo Scientific, Rockford, WA, USA) as described [46]. The probe sequences are listed in Table S1.

### 4.8. Dual-Luciferase Transcriptional Activity Assay

To construct the pOsTDC1-Luc, pOsTDC3-Luc, and pOsT5H-Luc plasmids, promoters of *OsTDC1*, *OsTDC3*, and *OsT5H* (~1.5 kb upstream of these genes) were amplified from Nipponbare and cloned into the modified pH2GW7 vector (PJG094) containing the firefly luciferase (fLUC) gene and the Renillaluciferase gene (rLUC) as reporters, while the full-length cDNA of *OsbZIP18* was cloned into the PJF754 (pEAQ-HT-DEST2) vector as an effector. The plasmids were transferred into *A. tumefaciens* EHA105 by electroporation and co-infiltrated into *N. benthamiana* leaves. The luciferase activities were measured using the Dual-Luciferase Reporter Assay System (Promega, Madison, WI, USA) according to the manufacturer’s instructions, and the LUC activity was normalized to the REN activity.

### 4.9. Statistical Analysis

The data were analyzed using Microsoft Office Excel 2010 and SPSS 23.0 (SPSS, IBM, Chicago, IL, USA). The results are expressed as means ± SD of at least three independent experiments. The differences among groups were determined using a Tukey’s test or a one-way ANOVA.

## 5. Conclusions

Serotonin plays an important role in various developmental processes and biotic/abiotic stress responses. However, the mechanisms of the involvement of serotonin in UV-B stress are largely unknown in plants, especially in crops. Here, we reported the identification of the first, to our knowledge, regulator of serotonin biosynthesis and revealed a regulated mechanism in controlling UV-B stress-responsive serotonin accumulation in plants. Our results bring new insights into the transcriptional regulation of serotonin biosynthesis and provide new clues for further investigation of its developmental and environmental regulation.

## Data Availability

All data are available upon reasonable request.

## Supplementary Materials

The following supporting information can be downloaded at: https://www.mdpi.com/article/10.3390/ijms23063215/s1.
