# Supplementary material for: OsbZIP18, a Positive Regulator of Serotonin Biosynthesis, Negatively Controls the UV-B Tolerance in Rice
Source: Int J Mol Sci. 2022 Mar 16;23(6):3215. doi: 10.3390/ijms23063215 (PMC8949417; doi:10.3390/ijms23063215)
Supplement: Supplementary file 1 [file ijms-23-03215-s001.zip › Supplementary Figures.pdf]

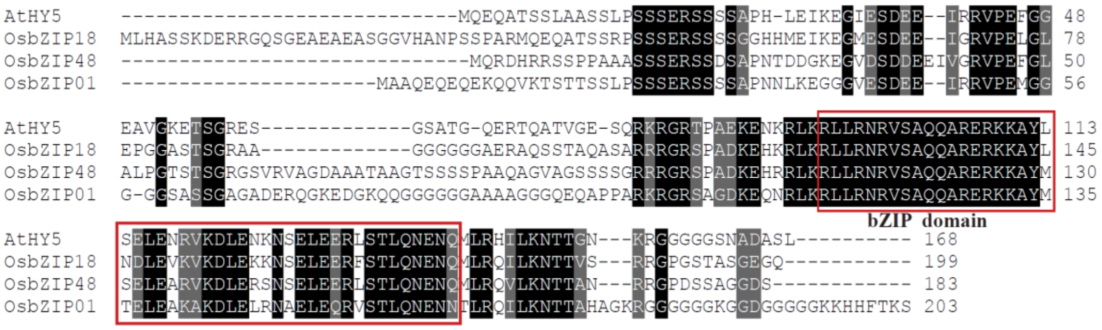

Supplementary Figure S1. Protein sequence alignment of HY5 in *Arabidopsis* and rice. The zinc-finger domain is boxed.

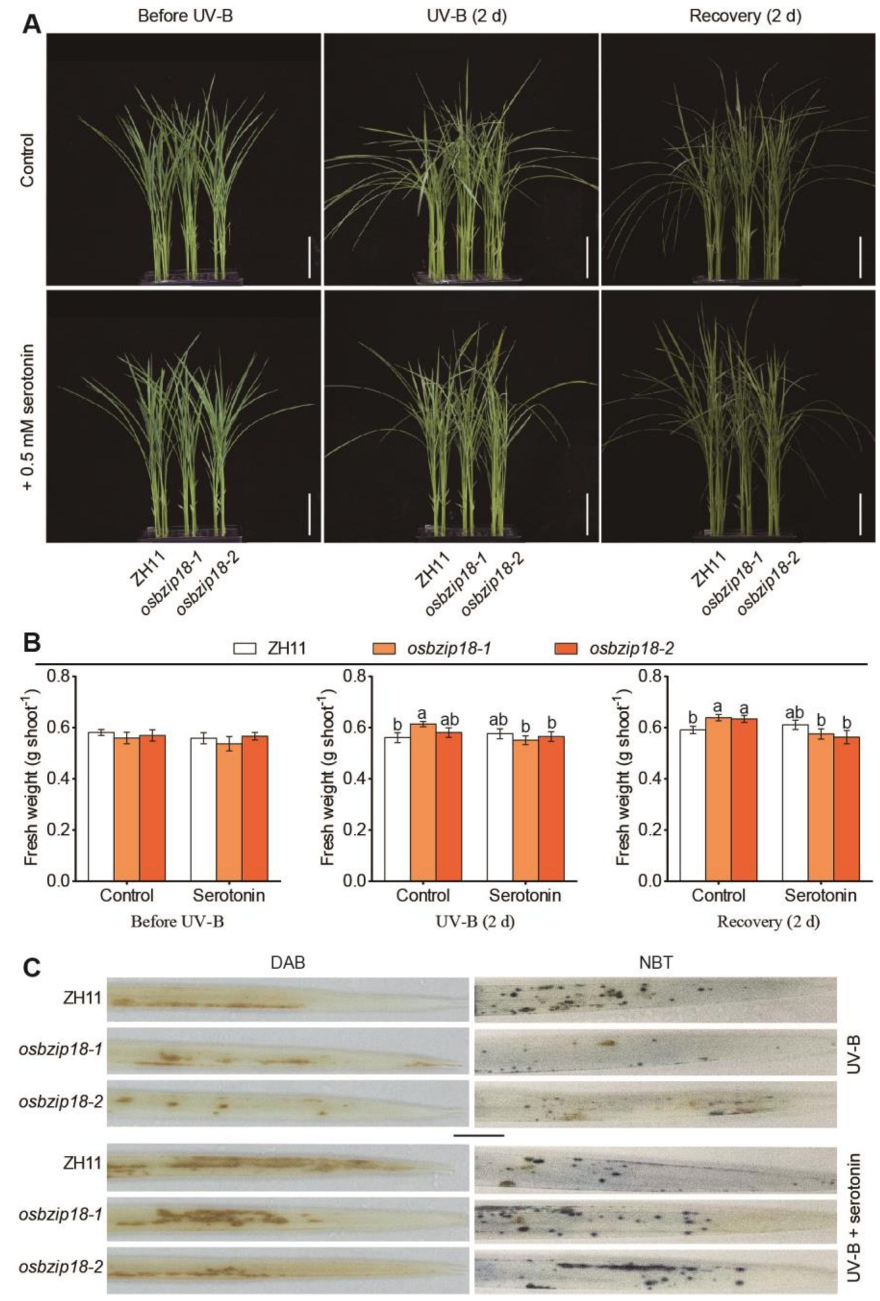

Supplementary Figure S2. Serotonin attenuates the UV-B stress-tolerance phenotypes of *osbzip18* mutants.

A, Phenotype comparison of *osbzip18* mutants before UV-B treatment, UV-B treatment for 2 d and after recovery from the UV-B treatment, which were grown in a nutrient solution with or without 0.5 mM serotonin. Plants were pretreated with 0.5 mM serotonin for 24 h before UV-B treatment. Scale bars=5 cm. B, The fresh weight of shoots in (A). Data are means ± SD of nine biological replicates. Different letters indicate significant difference at $P < 0.05$. C, Accumulation of ROS in the leaves treated with UV-B or UV-B + serotonin for 36 h. Scale bars=5 mm.
